# Predictors of Major Bleeding and Mortality in Dengue Infection: A Retrospective Observational Study in a Tertiary Care Centre in South India

**DOI:** 10.1155/2019/4823791

**Published:** 2019-09-04

**Authors:** Kevin John John, Karthik Gunasekaran, John Davis Prasad, Divya Mathew, Sohini Das, N. Sultan, Asha Mary Abraham, Ramya Iyyadurai

**Affiliations:** ^1^Department of Medicine, Christian Medical College, Vellore 632004, Tamil Nadu, India; ^2^Department of Clinical Virology, Christian Medical College, Vellore 632004, Tamil Nadu, India

## Abstract

We conducted a retrospective observational study to describe the clinical profile and outcomes of patients admitted with a diagnosis of dengue fever in a tertiary hospital in South India. A total of 159 patients admitted from April 2014 to October 2018 were included in the study. Vomiting (70.4%), myalgia (60.4%), headache (42.1%), abdominal pain (38.4%), bleeding (38%), and rash (37.1%) were the most common symptoms at presentation. The mean duration of hospital stay was 4.9 days (SD ± 2.4), and the median cost was INR 19,708 ($285) (IQR INR 12,968–32,056 ($188–$305)). Major bleeding was associated with elevated SGOT and SGPT, severe dengue, and secondary dengue. Mortality was associated with elderly age; elevated total leukocyte count, serum bilirubin, serum creatinine, SGOT, and SGPT; and high SOFA score. In view of these observations, we recommend stratifying patients according to the WHO classification of dengue and avoiding the use of thrombocytopenia as a single marker of the severity of the illness.

## 1. Introduction

Dengue fever is caused by the dengue virus, a member of the family Flaviviridae [1]. There are four antigenically distinct viruses in the dengue virus complex, namely, DENV-1, DENV-2, DENV-3, and DENV-4 [1]. Dengue is spread from human-to-human by the mosquito vector *Aedes aegypti* or *Aedes albopictus* during a blood meal [1].

In 1997, the World Health Organization (WHO) classified dengue infection into three types—dengue fever, dengue hemorrhagic fever, and dengue shock syndrome [2]. In 2009, this classification was revised, and the three types were identified as dengue without warning signs, dengue with warning signs, and severe dengue [3].

The diagnosis of dengue infection can be made at a very early stage using the reverse transcriptase polymerase chain reaction (RT-PCR) assay or detection of the nonstructural protein 1 (NS1) using the enzyme-linked immunosorbent assay (ELISA). The NS1 antigen may be detectable as early as day-1 postinfection and has a sensitivity of around 80% and specificity of 99% [4]. These tests have revolutionized the diagnosis of dengue.

Although there has been a substantial body of research on various aspects of dengue fever from around the world, there is a shortage of data from South India. Excessively large numbers of patients with dengue infection have been observed during certain months of the year, and the inadequacy of the health care system to cater to this patient load has often been acutely felt. Here, we present the clinical profile and outcomes of patients admitted with dengue infection in a tertiary hospital in South India over 5 years.

## 2. Materials and Methods

### 2.1. Study Design

This was a retrospective observational study of patients who were admitted to the hospital with a diagnosis of dengue fever as per the 2009 CDC (Centers for Disease Control and Prevention) case definition of dengue. The study was approved by the Institutional Review Board (IRB) before its commencement. The results are reported in accordance with the Strengthening the Reporting of Observational Studies in Epidemiology (STROBE) guidelines for reporting observational studies [5].

### 2.2. Setting

**Figure 1:**
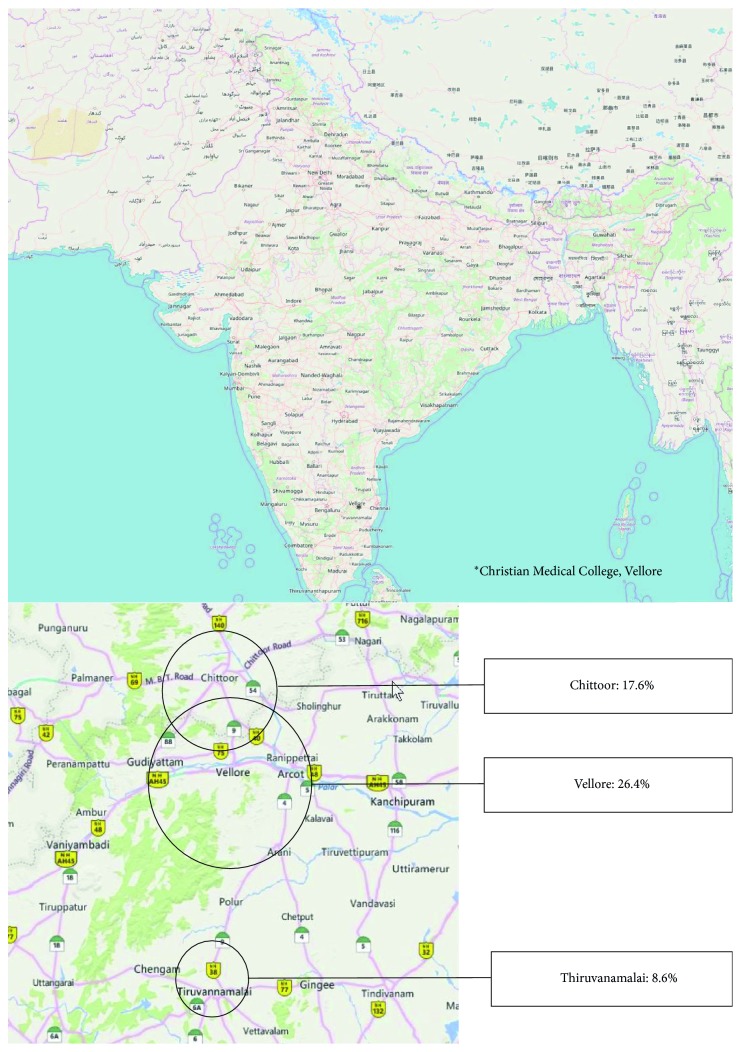
Location of Christian Medical College, Vellore, and distribution of cases in the districts surrounding Vellore.

This study was conducted in the Christian Medical College and Hospital, Vellore, a 2,858 bedded university teaching institute in South India (Figure 1). All patients who were admitted in the Department of Medicine (Unit-5) over a 5-year period, from April 2014 to October 2018, were included in the study.

### 2.3. Outcome Measures

Outcome measures that were looked at included mortality, major bleeding, need for intensive care, ventilation, dialysis, transfusion, organ system involvement, and cost of admission.

### 2.4. Statistical Methods

The patient data were extracted from electronic medical records, and analysis was done using SPSS 25 (IBM, Armonk, NY). All categorical baseline data was described using numbers and percentages. Continuous data were described using median and standard deviation. Cross tabulation was done with major bleed and mortality as outcome variables for various parameters. Statistically significant association with these outcomes was assessed using the Chi-square test, Fisher's exact test, and the independent samples *t*-test.

## 3. Results and Discussion

There were a total of 159 patients admitted with a diagnosis of dengue infection during the period under study, among whom there were 96 men (60.4%) and 63 women (39.6%). The mean age was 31.3 years (SD 13.5). There were 17 patients (10.7%) with diabetes mellitus and four patients (2.5%) on immunosuppressant medications.

Vomiting (70.4%), myalgia (60.4%), headache (42.1%), abdominal pain (38.4%), bleeding (38%), and rash (37.1%) were the most common symptoms at presentation (Table 1). The admission median hemoglobin was 144 g/L (interquartile range (IQR) 129–158), platelet count was 31 × 10^3^/*μ*L (IQR 15–85), WBC count was 4.9 × 10^9^/L (IQR 3.2–8.2), serum creatinine was 64.05 *μ*mol/L (IQR 53.4–78.5), total bilirubin was 0.6 mg/dL (IQR 0.4–0.9), SGOT (serum glutamic oxaloacetic transaminase) was 149 U/L (IQR 77–325), SGPT (serum glutamic pyruvic transaminase) was 92 U/L (IQR 37–189), and the mean SOFA (sequential organ failure assessment) score was 3.27 (Table 2). At presentation, hepatitis was present in 65.8%, encephalitis in 6.2%, and renal dysfunction in 5.6% of the patients, respectively.

Early dengue infection (NS1 antigen positive) was seen in 15.1% of patients, primary dengue (IgM antibody positive) in 23.9%, and secondary dengue (IgG antibody positive) in 76.2%.

About one-tenth of the patients who were admitted (12.6%) had dengue without warning signs, while 44% and 43.4% had dengue with warning signs and severe dengue, respectively, as per the WHO (World Health Organization) classification for severity of dengue.

The percentage of patients who received platelet transfusion was 27%, while those that received fresh frozen plasma and packed red cells were 19.5% and 1.2%, respectively.

Elevated SGOT and SGPT, severe dengue, and secondary dengue were associated with major bleed (Table 3). Increased age; elevated total leukocyte count, serum total bilirubin, serum creatinine, SGOT, and SGPT; and high SOFA (sequential organ failure assessment) score were associated with mortality (Table 4).

**Figure 2:**
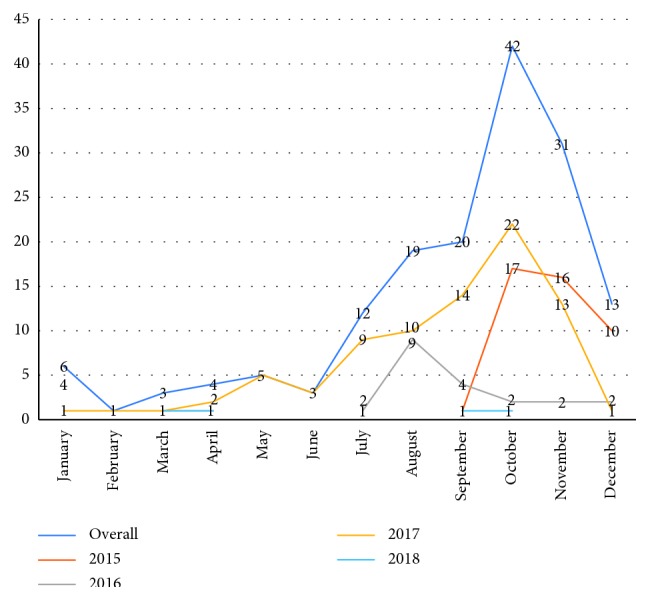
Monthly distribution of cases.

The maximum number of patients were admitted in October (26.4%), November (19.5%), September (12.6%), and August (12%) (Figure 2). Most patients were from Vellore (26.4%), Chittoor (17.6%), and Tiruvannamalai (8.6%) districts (Figure 1).

The mean duration of hospital stay was 4.9 days (SD 2.4), and the median cost was INR 19,708 ($285) (IQR INR 12,968–32,056 ($188–$305)). Four patients required ICU (intensive care unit) care―all four required ventilatory support, three required inotropic support, and one required hemodialysis. Eventually, all four patients died. Hence, the mortality rate for dengue infection in this study was four out of the total 159 patients (2.5%).

### 3.1. Discussion

Dengue fever is a major global public health problem, especially in the tropical and subtropical countries. The burden of the disease is particularly felt in countries like India, where, during the dengue “season,” the health care system is overwhelmed by patients with dengue fever. Not all patients who present to the hospital can be admitted for monitoring due to the finite number of beds. Hence, many patients are managed on an outpatient basis. Dengue is a unique disease in that the phase of the apparent resolution of illness with defervescence of fever may herald the progression into the critical phase, characterized by capillary leak, shock, multiorgan dysfunction, and bleeding. Although this occurs only in a small proportion of individuals, it is potentially fatal. The molecular and immunological mechanisms behind the propensity of specific individuals to progress to severe dengue have been elucidated with reasonable clarity [6]. However, from a clinical point of view, there are no reliable predictors of progression to severe dengue [7, 8].

In India, dengue is a notifiable disease in 14 states and is endemic in 29 states [9]. According to the annual report of the Department of Health and Family Welfare 2017-18, maximum cases were reported from Kerala [9]. Tamil Nadu, where this study was conducted, was third in the number of cases [9]. The total number of reported dengue cases has been increasing every year. This may be due to better case-reporting, rather than an actual increase in the incidence of the disease. There are also concerns that there is gross underreporting of cases, therefore underestimating the real burden of the disease. This is because the national figures are based on passive laboratory-based surveillance. One of the first population-based studies to estimate the incidence of dengue was conducted at Vellore and found an incidence of 49.5 cases per 1000 child years [10]. However, this study only included children. More data from the individual districts of India are unavailable at present.

The present study was done in a 2,858 bedded tertiary care teaching institute in South India with data from the past five years. The hospital caters to all strata of society. Although we are a referral center, we receive an unfiltered patient population from both the emergency department and outpatient services.

In our study, more men were affected by dengue fever than women, and the mean age was 31.3 years. Majority of the patients were thrombocytopenic at admission, and they had varying degrees of hepatic transaminase elevation. The most common organ involved was the liver, with hepatitis seen among 65.8% of the patients.

Most patients presented with secondary dengue (76.2%). Dengue with warning signs (44%) and severe dengue (43.4%) outnumbered patients with dengue without warning signs (12.6%). Only 1.2% of the patients had bleeding which required packed cell transfusion despite the significant incidence of thrombocytopenia. However, 27% of patients did receive platelet transfusions, and therefore, it cannot be ascertained if these patients would have bled if they had not been transfused. The maximum number of patients presented during the months of August to November, which corresponds to the monsoons in this part of the country. The mortality rate in the present study of 2.5% was comparable to the case fatality of 2.6%, reported in a systematic review and meta-analysis by Ganeshkumar et al. [11].

The modification of the 1997 classification of dengue fever by the WHO in 2009 was meant to help the physician in identifying patients with dengue fever who need intensive monitoring and resuscitation. However, the perception of dengue as a “thrombocytopenic” disease, the focus in the media on the associated bleeding and death as a consequence, has led to undue importance being given to the platelet count in the decision-making process. Often, it is the capillary leak and multiorgan dysfunction state that leads to death in dengue.

This is borne out by the data examined in the present study. Of the total number of patients admitted, more than one-tenth had dengue without warning signs, leading to the assumption that the admissions were for monitoring of the platelet count. Also, the median cost of admission was INR 19,708 ($285), which is by no means an insignificant amount, especially in a country like India where the per capita monthly income is INR 10,534 ($152). To put this into perspective, as of 2011, 21.9% of the population in India lives below the poverty line, and 21.2% of the employed population are below $1.9 in purchasing power parity per day. This cost is comparable to that reported from other studies in India and is the direct medical cost [11, 12]. The actual cost, which includes direct nonmedical costs and indirect costs, would be higher. Thus, a substantial cost saving could be made if unnecessary hospital admissions were avoided.

It should also be noted that there was no statistical association between thrombocytopenia and major bleeding. The factors that did predict major bleeding were SGOT, SGPT, severe dengue, and secondary dengue. A similar picture was seen with mortality—thrombocytopenia did not predict mortality, but elderly age; elevated total leukocyte count, serum bilirubin, serum creatinine, SGOT, and SGPT; and high SOFA score did. Dengue is usually associated with leukopenia. Hence, leukocytosis associated with dengue fever should be considered as a red flag.

These observations underscore the need to use the WHO classification of dengue fever while making decisions regarding whom to admit and follow-up on an outpatient basis. Although counterintuitive, looking at thrombocytopenia alone leads to unnecessary admissions in the hospital, undue financial burden to the patient, and the consumption of precious finite health care resources. However tempting the concept of prophylactic platelet transfusion in patients with thrombocytopenia may be, the benefits are nonevident. In fact, a multicenter, randomized control trial showed that prophylactic platelet transfusion was not superior to supportive care alone in preventing bleeding and that it might be associated with adverse effects [13].

Lastly, it was noted that all the patients who required intensive care succumbed to the illness. This emphasizes the need to provide aggressive resuscitation when patients present with warning signs and with severe dengue before they progress to the multiorgan dysfunction state. The steep rise in mortality at this stage has implications while explaining prognosis to the patient's relatives.

In conclusion, we recommend stratifying patients according to the WHO classification of dengue fever. We recommend avoiding the use of thrombocytopenia as a marker of the severity of the illness. Patients with dengue without warning signs may be managed on outpatient basis, while patients with dengue with warning signs, severe dengue, elderly patients, those with elevated leukocyte count, serum bilirubin, creatinine, SGOT, and SGPT, and high SOFA score should be preferably managed as in-patients, as they are at higher risk for major bleeding and death.

Dengue fever continues to be a significant public health problem in India and the world over. Various treatment strategies have been tried for dengue fever, including measures to increase platelet count [14]. With the progress in dengue vaccine research, it may soon become a vaccine-preventable disease [15–17]. However, until that becomes a reality we need to adapt and overcome and continually strive to better understand the disease and change practices according to the latest evidence.

## 4. Conclusions

In our study, young males more commonly presented with dengue infection. Most patients had nonspecific symptoms such as vomiting and myalgia, and 44.7% of patients had bleeding manifestations. The highest number of patients presented in October and was from the Vellore district. The mean SOFA score was 3.27, suggesting they were not very sick. Most patients had secondary dengue infection. Hepatitis was the most common complication of dengue infection. Most patients either had dengue with warning signs or severe dengue infection, as per the WHO classification for severity of dengue. Elevated SGOT and SGPT, severe dengue, and secondary dengue were associated with major bleeding. Increasing age; elevated total leukocyte count, serum total bilirubin, serum creatinine, SGOT, and SGPT; and high SOFA score were associated with increased mortality. The mortality in our study was 2.5%.

## Figures and Tables

**Table 1:** Presenting symptoms.

Symptoms	Number (%)
Vomiting	112 (70.4)
Myalgia	96 (60.4)
Headache	67 (42.1)
Major bleeding^*∗*^	51 (32.1)
Abdominal pain	61 (38.4)
Rash	59 (37.1)
Cough	39 (24.5)
Diarrhea	32 (20.1)
Minor bleeding	20 (12.6)
Arthralgia	20 (12.6)
Dyspnea	17 (10.7)
Altered mental status	10 (6.3)
Icterus	6 (3.8)

^*∗*^Major bleeding includes hematemesis, melena, hematochezia, hemoptysis, and hematuria.

**Table 2:** Lab investigations.

Lab investigation	Median value (IQR^*∗*^)
Hemoglobin (g/L)	144 (129–158)
Platelet count (×10^3^/*μ*L)	31 (15–85)
Total leukocyte count (×10^9^/L)	4.9 (3.2–8.2)
Serum creatinine (*μ*mol/L)	64.05 (53.4–78.5)
Total bilirubin (*μ*mol/L)	10.3 (6.8–15.4)
SGOT (U/L)	149 (77–325)
SGPT (U/L)	92 (37–189)

^*∗*^IQR, interquartile range.

**Table 3:** Predictors of major bleed.

Outcome	Major bleed		*p* value
	Yes	No	
Age^§^	31.5 (14.3)	31.3 (13.1)	0.93
Male (*n* = 96)	34	62	0.30
Days of fever^§^	5 (1.9)	5.14 (2.3)	0.71
Rash (*n* = 59)	17	42	0.498
Myalgia (*n* = 96)	26	70	0.96
Arthralgia (*n* = 20)	5	15	0.46
Vomiting (*n* = 112)	38	74	0.44
Loose stools (*n* = 32)	11	21	0.755
Abdominal pain (*n* = 61)	25	36	0.058
Platelet count (×10^3^/*μ*L)^§^	44 (57)	65 (67)	0.059
SGOT (serum glutamic oxaloacetic transaminase) (U/L)^§^	766 (2462)	254 (400)	0.036^*∗∗*^
SGPT (serum glutamic pyruvic transaminase) (U/L)^§^	343 (871)	139 (192)	0.021^*∗∗*^
SOFA^a^ score^§^	3.49 (1.6)	3.16 (1.9)	0.475
Late dengue^*∗∗∗*^ (*n* = 39)	13	26	0.283
Severe dengue (*n* = 69)	48	21	0.0001^*∗∗*^
Secondary dengue^⋄^ (*n* = 121)	45	76	0.014^*∗∗*^

^a^SOFA, sequential organ failure assessment; ^§^mean (standard deviation); ^*∗∗*^*p* value < 0.05 indicates a statistically significant association; ^*∗∗∗*^late dengue indicates presentation after 5 days of symptom onset; ^⋄^secondary dengue has both IgG and IgM antibodies positive, while primary dengue has IgM and/or NS1 antigen positive.

**Table 4:** Predictors of mortality.

Outcome	Mortality		*p* value
	Yes	No	
Age^§^	47.3 (18.7)	30.9 (13.1)	0.016^*∗∗*^
Male (*n* = 96)	2	94	0.649
Days of fever^§^	4.3 (2.2)	5.1 (2.2)	0.436
Rash (*n* = 59)	1	58	1.00
Myalgia (*n* = 96)	2	94	0.649
Arthralgia (*n* = 20)	1	19	0.419
Vomiting (*n* = 112)	3	109	1.00
Loose stools (*n* = 32)	0	32	0.584
Abdominal pain (*n* = 61)	2	59	0.638
Total leukocyte count (×10^9^/L)^§^	10.4 (5)	5.9 (3.6)	0.015^*∗∗*^
Platelet count (×10^3^/*μ*L)^§^	33 (36)	59 (65)	0.424
Creatinine (*μ*mol/L)^§^	109 (61)	67.9 (29)	0.009^*∗∗*^
Total bilirubin (*μ*mol/L)^§^	37.1 (22.2)	14.9 (20.5)	0.029^*∗∗*^
SGOT (serum glutamic oxaloacetic transaminase) (U/L)^§^	4831 (7833)	304 (652)	0.0001^*∗∗*^
SGPT (serum glutamic pyruvic transaminase) (U/L)^§^	1768 (2495)	164 (309)	0.0001^*∗∗*^
SOFA^a^ score^§^	7.25 (3.4)	3.16 (1.6)	0.0001^*∗∗*^
Major bleed (*n* = 108)	2	49	0.594
Late dengue^*∗∗∗*^ (*n* = 39)	13	26	1.00
Severe dengue (*n* = 69)	2	67	1.00
Secondary dengue^⋄^ (*n* = 121)	4	117	0.573

^a^SOFA, sequential organ failure assessment; ^§^mean (standard deviation); ^*∗∗*^*p* value < 0.05 indicates a statistically significant association; ^*∗∗∗*^late dengue indicates presentation after 5 days of symptom onset; ^⋄^secondary dengue has both IgG and IgM antibodies positive, while primary dengue has IgM and/or NS1 antigen positive.

## Data Availability

The data used to support the findings of this study are available from the first author and corresponding author upon request.
